# A modern reinterpretation of the “kidney governs bone” theory: systemic regulation of the neuro-endocrine-immune network via the brain-bone axis and traditional chinese medicine intervention strategies

**DOI:** 10.3389/fphar.2026.1862657

**Published:** 2026-07-29

**Authors:** Jiahao Dong, Zhongyu Peng, Tao Chen

**Affiliations:** 1 The First Clinical Medical College, Yunnan University of Chinese Medicine, Kunming, China; 2 Yunnan Provincial Hospital of Chinese Medicine, Kunming, China

**Keywords:** bone repair, brain-bone axis, kidney governs bone, neuro-endocrine-immune network, systemic regulation, traditional Chinese medicine

## Abstract

The traditional Chinese medicine (TCM) theory of “Kidney Governs Bone' has been progressively validated in modern medical research. However, a purely endocrinological perspective cannot fully explain its regulatory implications under complex conditions such as stress, inflammation, and nerve injury. As bone metabolism research shifts toward a systemic regulatory paradigm, the “brain-bone axis' provides a new framework for reinterpreting “Kidney Governs Bone.' Based on current evidence, the core scientific proposition of this article is that we propose the hypothesis that the therapeutic effects of TCM’s “Kidney Governs Bone' theory can be partially explained by systemic regulation of the neuro-endocrine-immune network, and that the therapeutic efficacy of TCM interventions may be related to their ability for layered, multi-target regulation within this network. This proposition requires further validation in diverse pathological contexts. Based on this proposition, we propose three conceptual frameworks: ① extending “Kidney Governs Bone' from the traditional “kidney-bone' endocrine axis to a systemic regulatory framework centered on the “brain-bone axis'; ② constructing a three-level “central-peripheral-local' regulatory model that systematically integrates the hierarchical interactions within the neuro-endocrine-immune network; and ③ proposing a “stratified intervention' strategy for TCM, clarifying the target differences among different therapeutic principles across the three network levels. Using this framework, we systematically evaluate the mechanisms of TCM interventions: kidney-tonifying herbs regulate the hypothalamic-pituitary axis to maintain endocrine homeostasis; kidney-tonifying and blood-activating methods, as well as acupuncture and other external therapies, achieve “neuro-vascular-osteogenic' synergy by modulating sympathetic tone and upregulating neuropeptides such as CGRP; acupuncture and moxibustion remodel the bone immune microenvironment by activating the cholinergic anti-inflammatory pathway, promoting M2 macrophage polarization, and inhibiting NLRP3 inflammasome activation. Current challenges include spatiotemporal heterogeneity, network complexity, and clinical translation gaps. Future studies should employ technologies such as optogenetics, multi-omics integration, and organoid-on-a-chip platforms. The modern scientific connotation of “Kidney Governs Bone' is deeply reflected in the systemic regulation of the neuro-endocrine-immune network, and this interdisciplinary direction provides a crucial theoretical breakthrough for transforming TCM orthopedics from empirical medicine toward systems medicine.

## 1 Introduction: theoretical paradigm shift from the “kidney-bone axis” to the “brain-bone axis”

The traditional Chinese medicine theory of “Kidney Governs Bone” holds that kidney function is closely related to bone health: sufficient kidney essence ensures strong bones, while kidney deficiency predisposes to osteoporosis, delayed fracture healing, and other bone disorders. This theory has gradually gained scientific validation in modern research, particularly in the pathological mechanisms of chronic kidney disease (CKD) and osteoporosis (OP), where the kidney-bone interaction—the “renal-osseous axis”—has become an important research area. Clinical observations show that CKD patients often present with CKD-mineral and bone disorder (CKD-MBD), characterized by low bone mineral density, high fracture risk, and abnormal bone turnover markers (e.g., elevated iPTH and FGF23, decreased 25-(OH)-vitamin D) (Aguilar et al., 2023; Kang et al., 2024), which highly aligns with the TCM concept of “kidney deficiency leading to bone weakness.” Moreover, the key roles of kidney-secreted Klotho protein and FGF23 in calcium-phosphate metabolism, together with their aberrant expression in diabetic kidney disease (DKD)-related bone metabolic disorders (Kang et al., 2024), further provide a molecular interpretation for “Kidney Governs Bone.”

In recent years, bone metabolism research has undergone a profound paradigm shift from “local self-repair” to “systemic regulation,” and the introduction of the “brain-bone axis” concept is a milestone in this transition. Traditional theories centered on Frost’s “mechanostat” concept, emphasizing that bone tissue maintains the balance between bone formation and resorption by sensing local mechanical stimuli (Wilzman et al., 2025; Frost, 1987). However, emerging evidence indicates that bone is not merely a passive target organ for mechanical adaptation but an active regulator that engages in bidirectional dialogue with the central nervous system via the neuro-endocrine-immune network. This conceptual renewal stems from three breakthroughs: bone-derived hormones (e.g., osteocalcin) can directly act on hypothalamic neurons to regulate energy metabolism and cognitive function (Hashimoto et al., 2025); the gut microbiota significantly influences bone remodeling through the “gut-brain-bone axis”-mediated immune modulation (Zhang et al., 2023); and the positive correlation between neuropsychological states (e.g., depression, insomnia) and bone disease risk reveals the remote regulatory potential of psycho-neural factors (Luo et al., 2023).

### 1.1 Botanical authentication of herbal materials

The following medicinal materials mentioned in this review are derived from authenticated botanical and zoological species (nomenclature verified against the Medicinal Plant Names Services [MPNS] database (MPNS, 2026) and Plants of the World Online [POWO] (POWO, 2026); full taxonomic details with part used and pharmacopoeial references are provided in Supplementary Table S1):

Epimedium brevicornu Maxim (Berberidaceae) — Herba Epimedii (Yinyanghuo).

Drynaria fortunei (Kunze) J. Sm (Polypodiaceae) — Drynariae Rhizoma (Gusuibu).

Rehmannia glutinosa (Gaertn.) DC (Orobanchaceae) — Rehmanniae Radix Praeparata (Shudihuang).

Cornus officinalis Siebold & Zucc (Cornaceae) — Corni Fructus (Shanzhuyu).

Dioscorea opposita Thunb (Dioscoreaceae) — Dioscoreae Rhizoma (Shanyao).

Lycium barbarum L (Solanaceae) — Lycii Fructus (Gouqizi).

Cuscuta chinensis Lam (Convolvulaceae) — Cuscutae Semen (Tusizi).

Achyranthes bidentata Blume (Amaranthaceae) — Achyranthis Bidentatae Radix (Niuxi).

Eucommia ulmoides Oliv (Eucommiaceae) — Eucommiae Cortex (Duzhong).

Cinnamomum cassia (L.) J. Presl (Lauraceae) — Cinnamomi Ramulus (Guizhi).

Aconitum carmichaelii Debeaux (Ranunculaceae) — Aconiti Lateralis Radix Praeparata (Fuzi).

Angelica sinensis (Oliv.) Diels (Apiaceae) — Angelicae Sinensis Radix (Danggui).

Salvia miltiorrhiza Bunge (Lamiaceae) — Salviae Miltiorrhizae Radix et Rhizoma (Danshen).

Ligusticum striatum DC (Apiaceae) — Chuanxiong Rhizoma (Chuanxiong).

*Cervus elaphus* Linnaeus (Cervidae) — Cervi Cornus Colla (Lujiaojiao).

Mauremys reevesii (Gray) (Geoemydidae) — Testudinis Carapacis et Plastri Colla (Guibanjiao).

All herbal materials cited in this review correspond to the authenticated species listed in the Chinese Pharmacopoeia (2020 edition). For compound formulas (Zuogui Pill and Yougui Pill), the full compositional details with botanical origins are provided in Supplementary Table S1. All botanical nomenclature followed the recommendations of Rivera et al. (2014) for accurate scientific naming of medicinal plants.

### 1.2 Theoretical framework and scope of this review

Before presenting the three-level regulatory model, we must explicitly delineate the theoretical boundaries and epistemological status of this framework.

First, the “brain-bone axis” concept has been previously established in the literature (e.g., Ryu et al. (2024)); the innovation of this review does not lie in proposing this concept *de novo*, but in (i) mapping the classical TCM “Kidney Governs Bone” theory onto the three functional levels of this axis, and (ii) systematically proposing differential target specificities of distinct TCM therapeutic principles (kidney-tonifying, blood-activating, acupuncture/manipulation) within this axis—a differentiation that, to our knowledge, has not been previously articulated.

Second, we acknowledge that the three-level model presented herein represents an inductive synthesis of existing fragmented evidence rather than a deductively validated theory. Its status is that of a working hypothesis that generates the following testable predictions:

Prediction 1: Different TCM therapeutic principles should exert dominant effects at distinct levels of the neuro-endocrine-immune network—kidney-tonifying herbs primarily at the central endocrine level (hypothalamic-pituitary axes), acupuncture/manipulation primarily at the peripheral sensory nerve terminal level (CGRP/SP release), and blood-activating herbs primarily at the local neuro-immune interface (macrophage polarization).

Prediction 2: If a therapeutic principle is applied at a non-corresponding stage of bone repair (e.g., kidney-tonifying herbs during the acute hematoma phase), its therapeutic efficacy should be significantly diminished compared to stage-matched intervention.

Prediction 3: Combinatorial therapy targeting multiple levels of the network simultaneously (e.g., kidney-tonifying herbs + acupuncture) should yield synergistic effects greater than the sum of individual interventions.

Third, we recognize a fundamental epistemological challenge: TCM is a philosophical and empirical system with its own internal logic that cannot be fully reduced to biomedical pathways. Our approach is not to claim that “Kidney Governs Bone” equals any specific molecular pathway, but rather to propose that the observable therapeutic effects of TCM interventions classified under this theory can be partially explained by their modulation of the neuro-endocrine-immune network. This is a translational bridge rather than a reductionist equivalence.

Fourth, the generalizability of this three-level framework across different pathological conditions (acute vs. chronic bone injuries, inflammatory vs. non-inflammatory bone diseases, young vs. aging populations) requires systematic testing in future studies. The current review focuses on fracture healing and osteoporosis as primary exemplars, and extrapolation to other bone pathologies should be made with caution.

**FIGURE 1:**
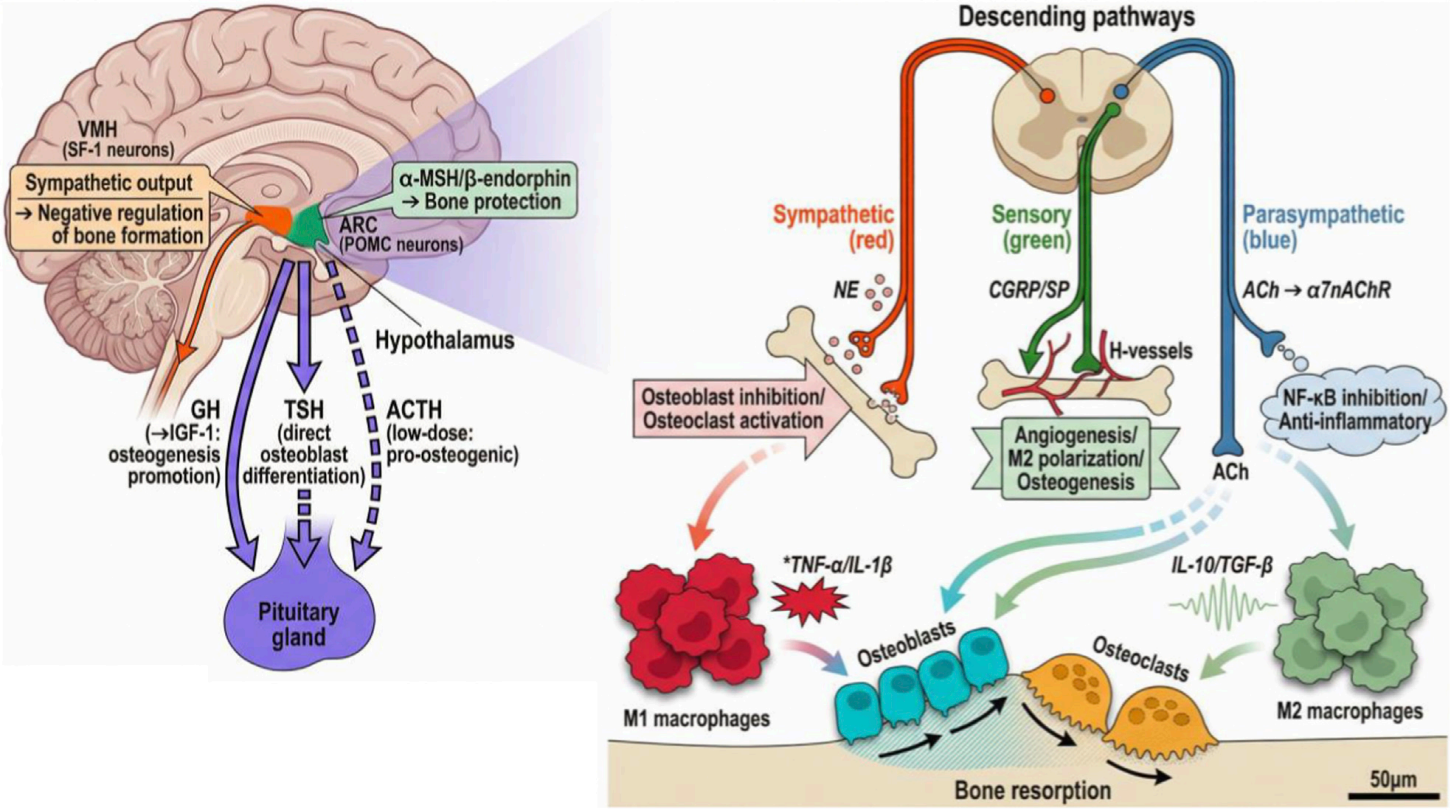
Schematic illustration of the three-level regulatory network constituting the “brain-bone axis”. Top (central hub): Hypothalamic nuclei (SF-1^+^ neurons in the ventromedial hypothalamus, VMH; POMC^+^ neurons in the arcuate nucleus, ARC) and pituitary hormones (GH, TSH, ACTH) integrate systemic signals. Middle (descending pathways): Sympathetic (red, NE/β2-AR), sensory (green, CGRP/SP), and parasympathetic (blue, ACh/α7nAChR) nerves transmit signals from the central nervous system to peripheral bone tissues. Bottom (effector): Bone immune microenvironment, where neurotransmitters and neuropeptides regulate macrophage polarization, T cell subsets, and osteoblast/osteoclast activity, collectively determining bone formation and resorption balance. Abbreviations: SF-1, steroidogenic factor-1; POMC, pro-opiomelanocortin; GH, growth hormone; TSH, thyroid-stimulating hormone; ACTH, adrenocorticotropic hormone; NE, norepinephrine; β2-AR, beta-2 adrenergic receptor; CGRP, calcitonin gene-related peptide; SP, substance P; ACh, acetylcholine; α7nAChR, alpha 7 nicotinic acetylcholine receptor; OB, osteoblast; OC, osteoclast. Abbreviations key: VMH, Ventromedial Hypothalamus; SF-1, Steroidogenic Factor 1, ARC, Arcuate Nucleus; POMC, Pro-opiomelanocortin; a-MSH, Alpha-Melanocyte-Stimulating Hormone; GH, Growth Hormone; IGF-1, Insulin-like Growth Factor 1; TSH, Thyroid-Stimulating Hormone; ACTH, Adrenocorticotropic Hormone; NE, Norepinephrine; ẞ2-AR, Beta-2 Adrenergic Receptor; CGRP, Calcitonin Gene-Related Peptide; SP, Substance P; ACh, Acetylcholine; a7nAChR, Alpha-7 Nicotinic Acetylcholine Receptor; TNF-a, Tumor Necrosis Factor Alpha; IL-18, Interleukin-1 Beta; IL-10, Interleukin-10; TGF-B, Transforming Growth Factor Beta; OB, Osteoblast; OC, Osteoclast; BMU, Bone Multicellular Unit.

**FIGURE 2:**
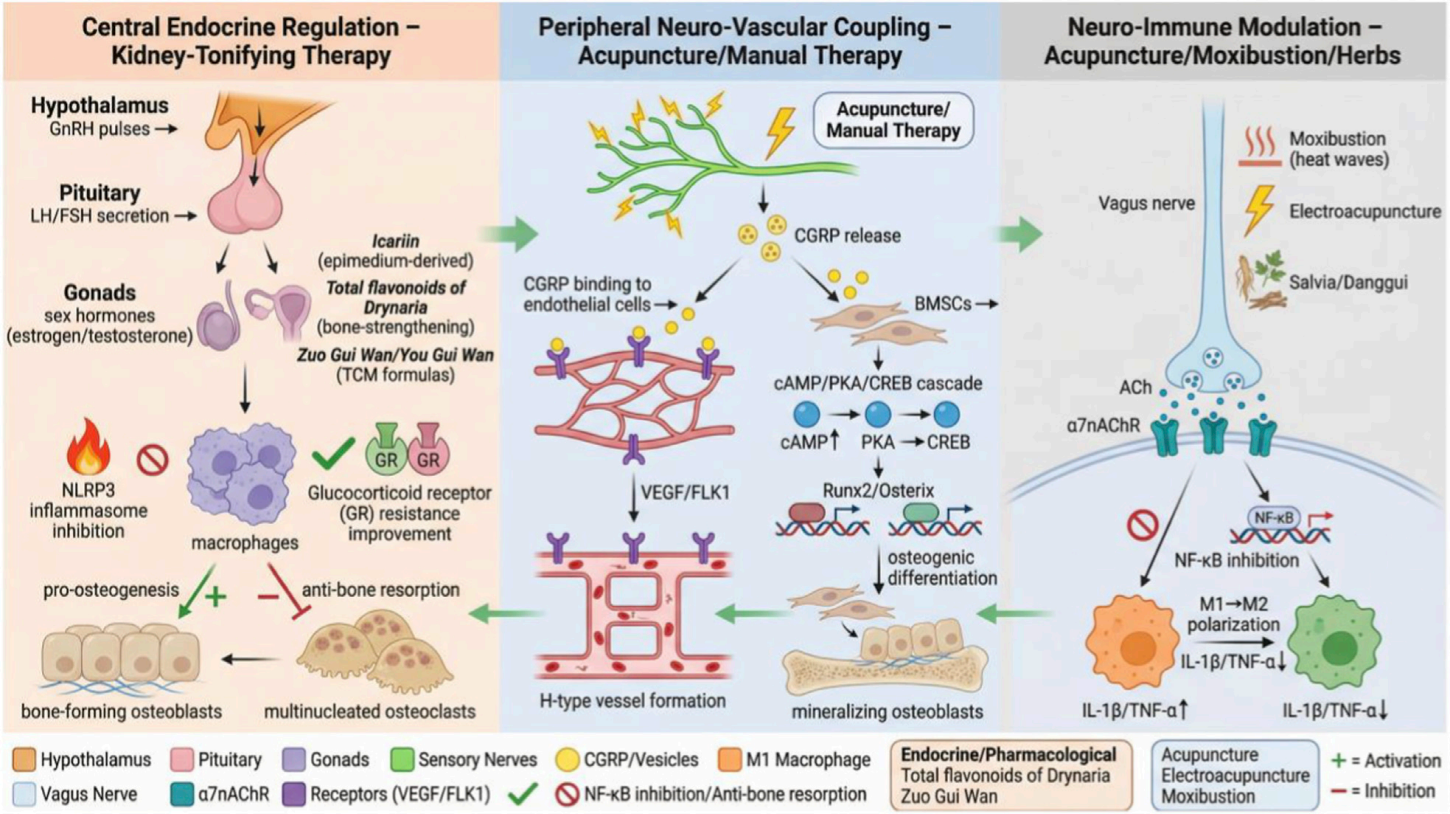
Schematic diagram illustrating the multi-target regulatory mechanisms of traditional Chinese medicine (TCM) interventions on the “brain-bone axis”. Left (central endocrine regulation--kidney-tonifying herbs): Herbs such as Epimedium brevicornu, Drynaria fortunei, and Zuogui/Yougui Pills modulate the hypothalamic-pituitary-gonadal/adrenal axes, regulating sex hormone levels and reducing glucocorticoid resistance via NLRP3 inflammasome inhibition. Middle (peripheral neuro-vascular coupling--acupuncture/manipulation): Physical stimulation activates sensory nerve terminals to release CGRP, which promotes H-type vessel formation via the VEGF pathway and enhances osteogenic differentiation of bone marrow mesenchymal stem cells (BMSCs) via the cAMP/PKA/CREB/Runx2 axis. Right (neuro-immune modulation--acupuncture/moxibustion/blood-activating herbs): Acupuncture and moxibustion activate the cholinergic anti-inflammatory pathway (CAP) through ACh/α7nAChR signaling, suppressing NF-κB activation, promoting M1-to-M2 macrophage polarization, and reducing pro-inflammatory cytokine release. Abbreviations: HPA, hypothalamic-pituitary-adrenal; HPG, hypothalamic-pituitary-gonadal; CGRP, calcitonin gene-related peptide; VEGF, vascular endothelial growth factor; BMSC, bone marrow mesenchymal stem cell; ACh, acetylcholine; α7nAChR, alpha 7 nicotinic acetylcholine receptor; NF-κB, nuclear factor kappa-B; IL, interleukin; TNF-α, tumor necrosis factor-alpha.

**FIGURE 3:**
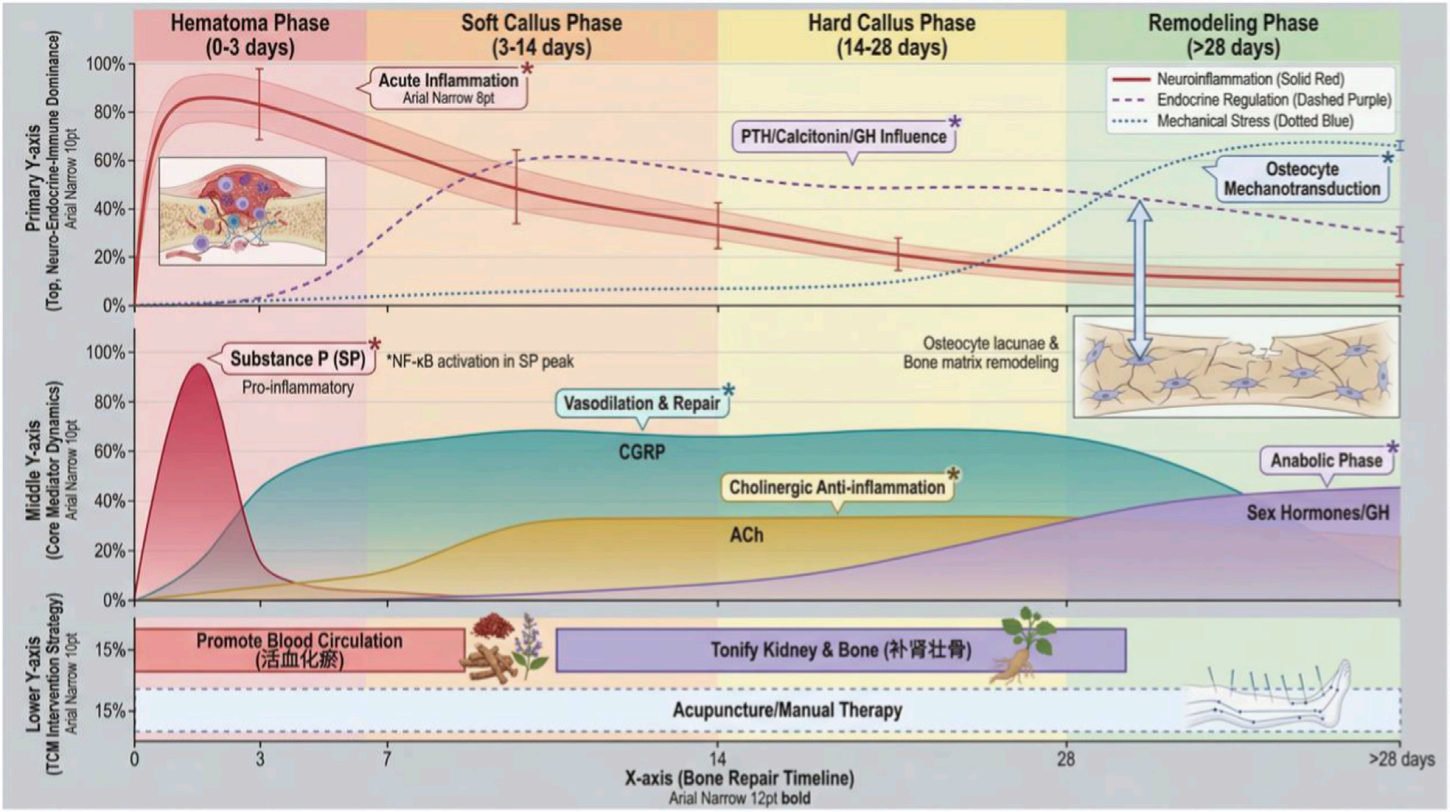
Temporal mapping of the dominant regulatory nodes within the neuro-endocrine-immune network across the four sequential phases of bone repair: hematoma phase (days 0–3), cartilaginous callus phase (days 3–14), hard callus phase (days 14–28), and bone remodeling phase (>28 days). Upper panel: Relative dominance of neuro-inflammatory responses (red), endocrine hormone regulation (purple), and neural remodeling/mechanical adaptation (blue) across the repair timeline. Middle panel: Dynamic changes in key neurotransmitters and hormones (SP, CGRP, ACh, sex hormones, GH). Lower panel: Optimal interventional windows for the three core TCM therapeutic principles. This temporal alignment illustrates the TCM principle of “treatment tailored to the phase of disease.” Abbreviations: SP, substance P; CGRP, calcitonin gene-related peptide; ACh, acetylcholine; GH, growth hormone.

Based on the above core scientific proposition—namely, that the therapeutic effects of TCM’s “Kidney Governs Bone” can be partially explained by systemic regulation of the neuro-endocrine-immune network—this paper aims to construct an interdisciplinary dialogue framework, positioning the traditional wisdom of “Kidney Governs Bone” within the modern systems biology network of “neuro-endocrine-immune” regulation, and to systematically evaluate the regulatory effects and potential targets of TCM in promoting bone repair through this network, thereby providing theoretical support for the modernization and internationalization of TCM orthopedics. To visually present this systemic regulatory network, we construct a “central-peripheral-local” three-level regulatory model (Figure 1), systematically map the target landscape of TCM interventions (Figure 2), and describe their spatiotemporal dynamic characteristics (Figure 3; Table 1).

**TABLE 1:** Targeted differences and clinical characteristics of three core therapeutic principles in traditional Chinese orthopedics within the “brain-bone axis” framework.

Therapeutic principle	Representative formulas/Herbs/Modalities	Primary target level	Key effector cells/Molecules	Functional characteristics	Optimal clinical stage	Evidence level*
Activating blood and removing stasis	Salvia miltiorrhiza, Ligusticum chuanxiong, Tao-Hong-Si-Wu decoction	Peripheral neuro-immune interface	Microglia, M1/M2 macrophages, TLR4/NF-κB pathway	Anti-inflammatory, alleviates neurotoxicity, hematoma clearance	Early stage (hematoma phase)	II-III (primarily animal and cellular mechanistic studies)
Tonifying kidney and strengthening bone	Epimedium brevicornu, drynaria fortunei, Zuogui Pill, Yougui Pill	Central endocrine axis (HPA/HPG axes)	Hypothalamic neurons, pituitary gland, sex hormone receptors, OPG/RANKL system	Long-term homeostatic regulation, matrix mineralization promotion	Middle to late stage (repair and remodeling phases)	I-II (limited high-quality RCTs directly validating “brain-bone axis” modulation; evidence primarily from animal models and a few clinical observational studies)
Acupuncture and manipulation	Electroacupuncture, moxibustion, manipulative reduction	Peripheral sensory nerve terminals	TRPV1 channel, CGRP/SP, α7nAChR, VEGF	Rapid onset, neuro-vascular-osteogenic coupling	All stages (analgesia and local microenvironment improvement)	II (clinical studies mainly on analgesia and functional outcomes; mechanistic links to “brain-bone axis” require further validation)

*Evidence levels are based on the authors’ assessment of the existing literature and do not derive from a formal GRADE or Cochrane systematic review. Grade I, meta‐analyses or well‐designed RCTs; Grade II, non‐randomized controlled trials, cohort, or case‐control studies; Grade III, animal or cellular mechanistic studies.

## 2 Scientific connotation of the “kidney governs bone” theory: from classical tracing to modern extension

### 2.1 Classical theoretical tracing: biological basis from “kidney stores essence” to “marrow nourishes bone”

In the theoretical system of traditional Chinese medicine, the core physiological mechanism of “kidney stores essence, essence generates marrow, and marrow nourishes bone” profoundly reveals the biological association between kidney function and bone metabolism. This theory holds that kidney essence, as the foundation of innate constitution, directly participates in bone growth, repair, and remodeling through the generation of bone marrow, a process that forms an interdisciplinary echo with key aspects emphasized in modern bone tissue engineering, such as cell differentiation, matrix mineralization, and vascularization (Wu et al., 2024). The late stage of fracture healing (i.e., the “liver-kidney tonifying stage” in the three-stage differentiation) is particularly dependent on sufficient kidney essence; clinical observations show that patients with kidney essence deficiency often exhibit delayed callus formation, reduced bone mineral density, and insufficient mechanical strength, overlapping with the mechanisms of osteoporotic fracture healing impairment (Steppe et al., 2023; Lin et al., 2025a). Syndrome-based treatment guided by this theory exhibits multi-target advantages: Zuogui Pills reduce oxidative stress and osteoblast apoptosis by regulating the Nrf2/HO-1 pathway, while upregulating osteoprotegerin (OPG) expression to improve bone microstructure (Lin et al., 2025a); Wuzi Yanzong Pills indirectly enhance bone formation by modulating sex hormone levels and antioxidant enzyme activity, providing a dual therapeutic strategy for male osteoporosis patients with fractures (Zhimin et al., 2023; Zha et al., 2026).

### 2.2 Evolution of modern interpretation: from endocrine axes to cross-organ networks

From the classical hypothalamic-pituitary-gonadal/thyroid axes (HPG/HPT axes) to the emerging “renal-osseous axis,” exploration of cross-organ signaling networks continues to deepen. The HPG axis regulates gonadal function via GnRH neurons; recent studies have found that microglia participate in GnRH neuronal synaptic pruning and functional regulation through the RANK signaling pathway (Collado-Sole et al., 2026; Mohammadzadeh and Amberg, 2023). The HPT axis regulates metabolism and development via thyroid hormone feedback. Both axes exhibit cross-regulation with bone homeostasis—sex hormone deficiency accelerates osteoporosis, while hyperthyroidism promotes bone resorption (González-Casaus, 2023; Bick et al., 2026). The proposal of the “renal-osseous axis” further expands the traditional endocrine framework, with its core molecules vitamin D and Klotho protein forming a bidirectional regulatory network: vitamin D is converted by renal 1α-hydroxylase into its active form 1,25(OH)_2_D_3_, which regulates intestinal calcium-phosphate absorption and directly acts on bone cells; Klotho, as an anti-aging protein, can both serve as a co-receptor for FGF23 in phosphate metabolism and independently regulate the Wnt/β-catenin pathway to influence bone formation (Knapp et al., 2025). CKD patients commonly exhibit vitamin D deficiency and downregulated Klotho expression, with compensatory elevation of FGF23, leading to secondary hyperparathyroidism and vascular calcification, forming a vicious cycle of “renal osteodystrophy-vascular calcification” (Zengwei et al., 2025).

### 2.3 Beyond endocrinology: the necessity of integrating neuro-immune dimensions

Although the endocrine regulatory mechanisms centered on the “renal-osseous axis” have deepened our understanding of the kidney-bone interaction, a purely endocrinological perspective still cannot fully explain the regulatory implications of “Kidney Governs Bone” under complex pathological conditions. Abnormal changes in bone metabolism under acute/chronic stress, local inflammatory microenvironment, and nerve injury often cannot be adequately explained by fluctuations in hormone levels. The excessive inflammatory response during the hematoma phase after fracture, the remote regulation of bone by the central nervous system, the direct influence of peripheral nerve-derived neurotransmitters on the bone immune microenvironment, and the dynamic balance of sympathetic-parasympathetic tone all play irreplaceable roles in bone repair. These lines of evidence suggest that the scientific connotation of “Kidney Governs Bone” should transcend the traditional “kidney-bone” endocrine axis and be re-examined within a more integrated “neuro-endocrine-immune” network. Accordingly, the following section will systematically elaborate the biological basis of the “brain-bone axis” to provide a new theoretical framework for understanding the systemic regulatory mechanisms of “Kidney Governs Bone.”

## 3 Biological basis of the “brain-bone axis”: a three-level architecture of the neuro-endocrine-immune network

### 3.1 Central hub: bidirectional regulation by the hypothalamic-pituitary axis

The hypothalamus, as the central hub for the central nervous system regulation of metabolism and endocrine function, has its neuronal subpopulations increasingly recognized for their roles in bone metabolism regulation. Steroidogenic factor-1 (SF-1) neurons and pro-opiomelanocortin (POMC) neurons exert bidirectional regulation of bone homeostasis through multi-level neuro-endocrine networks (Tadross et al., 2025). SF-1 neurons are mainly distributed in the ventromedial hypothalamus (VMH) and regulate the balance between bone formation and resorption via sympathetic output: their activation inhibits osteoblast activity and promotes osteoclast differentiation through the β2-adrenergic receptor pathway, forming a negative “nerve-bone axis” regulation (Beddows et al., 2024; Mei et al., 2023). This process is closely associated with energy metabolism, as SF-1 neurons can simultaneously adjust bone metabolic rate when sensing blood glucose changes, thereby achieving systemic coordination of energy allocation (Tadross et al., 2025; Beddows et al., 2024). POMC neurons are located in the arcuate nucleus (ARC) and exert bone-protective effects via neurotransmitters such as α-MSH and β-endorphin. Single-cell sequencing has revealed significant transcriptomic differences between human and mouse POMC neurons, suggesting that species specificity may influence their bone metabolism regulatory patterns (Tadross et al., 2025). It should be noted that the above-mentioned hypothalamic neuronal regulation of bone metabolism is largely based on animal experiments (especially rodents), and the significant transcriptomic differences between human and mouse POMC neurons (Tadross et al., 2025) warrant caution when directly extrapolating such mechanisms to human bone diseases. POMC neurons act through two parallel pathways: directly promoting osteoblast differentiation via melanocortin 4 receptor (MC4R), and indirectly attenuating sympathetic negative regulation of bone by inhibiting AgRP neurons (Alcantara et al., 2025; Fong et al., 2023).

The pituitary gland, as the central organ of the neuroendocrine system, finely regulates the dynamic balance of osteogenesis and osteoclastogenesis by secreting growth hormone (GH), thyroid-stimulating hormone (TSH), and adrenocorticotropic hormone (ACTH). GH promotes bone formation via insulin-like growth factor-1 (IGF-1) mediation, but its action is dual—stimulating osteoblast differentiation while enhancing osteoclast activity through the RANKL/OPG system; supraphysiological doses can lead to bone structural disorders, whereas physiological pulsatile secretion maintains normal bone mass (Halupczok-Żyła et al., 2024; Balci et al., 1992). TSH actions extend beyond the indirect effects of thyroid hormones: its receptor (TSHr) is expressed on both osteoblasts and osteoclasts, directly inhibiting adipogenesis and promoting mesenchymal stem cell differentiation toward the osteoblast lineage via the G protein/C/EBPβ signaling pathway (Huang J. et al., 2024; Winningham et al., 2025), while also participating in bone remodeling coupling by modulating TGF-β1 release (Chiba-Ohkuma et al., 2025; Wang and Luo, 2025). ACTH exhibits a unique biphasic regulation of bone metabolism: supraphysiological doses cause bone loss through glucocorticoid pathways, whereas very low sub-cortisol doses (1 pM-1 nM) significantly enhance osteogenic activity via MC2R activation of the VEGF/FLT-1 signaling axis, upregulating osteogenic markers such as Runx2 and Col1A1, and promoting mitochondrial complex I activity (Tourkova et al., 2025).

### 3.2 Descending pathways: sympathetic, sensory, and parasympathetic nerve conduction

The sympathetic nervous system (SNS) plays a complex and critical role in bone metabolism regulation, exerting bidirectional effects on osteoblast and osteoclast function via the norepinephrine (NE)-mediated β2-adrenergic receptor (β2-AR) signaling pathway. Studies have shown that excessive SNS activation significantly inhibits osteoblast differentiation and function via β2-AR, while indirectly promoting osteoclast activity, thereby disrupting the balance between bone formation and resorption, leading to bone loss and osteoporosis (Leroux et al., 2024; Ryu et al., 2024). This mechanism is particularly prominent under chronic stress, aging, and metabolic diseases (e.g., diabetes and obesity), where sustained SNS activation downregulates the expression of Runt-related transcription factor 2 (Runx2) and Osterix in osteoblasts via β2-AR, suppressing bone matrix synthesis (Ryu et al., 2024; Sakamoto et al., 2025). In addition, NE activates the cyclic AMP (cAMP)-protein kinase A (PKA) pathway in osteoclast precursors via β2-AR, promoting RANKL expression and thereby enhancing osteoclast differentiation and bone resorption (Leroux et al., 2024; Ryu et al., 2024).

In the molecular mechanisms of nerve-bone interaction, the spatiotemporally specific activation of β2-AR signaling is key. β2-AR on osteoblast membranes couples with Gαs proteins, and its activation inhibits Wnt/β-catenin signaling via the cAMP-PKA and ERK1/2 pathways, thereby weakening osteoblast proliferation and mineralization capacity (Ryu et al., 2024; Sakamoto et al., 2025). Conversely, β2-AR activation in osteoclasts and their precursors upregulates NFATc1 expression via cAMP response element-binding protein (CREB) phosphorylation; NFATc1 is a core transcription factor for osteoclast differentiation. This differential regulation suggests that β2-AR signaling exhibits significant cell-type specificity in bone tissue cells, possibly related to the differential recruitment of downstream effectors such as β-arrestin (Sakamoto et al., 2025).

The sensory nervous system plays a key role in bone metabolism, angiogenesis, and tissue repair by releasing neuropeptides such as calcitonin gene-related peptide (CGRP) and substance P (SP). Recent studies have revealed the regulatory effects of these neuropeptides on periosteal stem cells (e.g., dental pulp stem cells DPSCs and periodontal ligament cells) and the molecular mechanisms by which they enhance bone repair through promoting H-type vessel formation (Miki et al., 2024; Lynch et al., 2025). CGRP, a 37-amino acid neuropeptide secreted by sensory nerve endings, not only possesses potent vasodilatory functions but also directly stimulates endothelial cell proliferation and tube formation by upregulating vascular endothelial growth factor (VEGF) expression, thereby promoting angiogenesis (Leroux et al., 2024; Zhu et al., 2024). During bone repair, the spatiotemporal co-localization of CGRP + sensory nerve fibers with CD31^+^ or Emcn + blood vessels further confirms the importance of neuro-vascular coupling in bone regeneration (Han et al., 2023). Experiments have shown that CGRP promotes the collective migration of dental pulp stem cells to injury sites via the receptor activity-modifying protein 1 (RAMP1) pathway, significantly improving pulp repair quality (Wang C. et al., 2024). Moreover, CGRP also optimizes the local microenvironment and accelerates tissue healing by regulating macrophage polarization toward a pro-repair (M2) phenotype (Zhan et al., 2025).

Substance P (SP), as another key neuropeptide, exhibits both synergistic and differential effects compared to CGRP. SP activates via the neurokinin-1 receptor (NK1R), inducing a strong neurogenic inflammatory response during the acute inflammatory phase, but subsequently promotes tissue repair by shortening the inflammatory phase and facilitating M2 macrophage polarization (Cil et al., 2026; Siddiqui et al., 2023). In angiogenesis, sensory nerves exert a central role through the TRPV1-CGRP signaling axis. TRPV1, as a nociceptive receptor, not only upregulates CGRP release upon activation but also enhances endothelial cell function via the PI3K/Akt/CaMKII pathway, increasing vessel volume, number, and thickness (Leroux et al., 2024; Zhu et al., 2024). H-type vessels (CD31+Emcn+), as a bone-specific vascular subtype, exhibit a high spatiotemporal correlation with CGRP expression, and Sema3A (a nerve-secreted guidance molecule) may promote mature vascular network formation by regulating neuro-vascular interactions (Lynch et al., 2025; Han et al., 2023; Kamm et al., 2025).

The parasympathetic nervous system (PNS) plays a key role in bone immune microenvironment regulation via the cholinergic anti-inflammatory pathway (CAP). The core of CAP is that the vagus nerve releases acetylcholine (ACh), which acts on α7 nicotinic acetylcholine receptors (α7nAChR) to inhibit the release of pro-inflammatory cytokines (e.g., TNF-α, IL-1β, and HMGB1) from macrophages, thereby forming a neuro-immune-bone three-dimensional regulatory network (Cheng et al., 2025; Bingül et al., 2024; Ma et al., 2025). Under chronic stress, excessive SNS activation promotes osteoclast differentiation via β2-AR signaling, while PNS activation can antagonize this process, resulting in reduced bone resorption and increased bone mass. For example, chemical sympathectomy or β-blocker (e.g., propranolol) administration significantly improves chronic stress-induced osteoporosis, while vagus nerve stimulation (VNS) inhibits inflammatory bone destruction via an α7nAChR-dependent pathway (Xu R. et al., 2024).

### 3.3 Terminal effector: multi-cellular integrative regulation of the bone immune microenvironment

Neurotransmitters, as core mediators of inter-neuronal communication, have extended their scope beyond traditional synaptic transmission to regulate peripheral immune cell functions via paracrine, volume transmission, and extracellular vesicle pathways. The bone immune microenvironment, as the terminal effector of the “brain-bone axis,” integrates multiple neural signals from the central and peripheral systems, ultimately achieving systemic regulation of bone metabolism by the neuro-endocrine-immune network through the modulation of macrophage polarization, T cell subset balance, and the fate of osteoblast/osteoclast precursors.

In terms of macrophage polarization regulation, vesicles released by dopaminergic neurons can deliver miR-155 to macrophages via extracellular vesicles (EVs), promoting M1 polarization by inhibiting the TGF-β pathway; whereas M2 polarization is regulated by transcriptional activation of serotonin receptor 2B (HTR2B) (Picco et al., 2024; Bose et al., 2024). The transcription factor SP1 directly binds to the HTR2B promoter region, simultaneously enhancing M1 marker (iNOS, CD86) expression and suppressing M2 marker (Arg-1, CD206) expression, a dual regulation that exacerbates neuroinflammation in spinal cord injury models (Bose et al., 2024). In addition, ATP released from synaptic vesicles activates the CCL3/CCR1 signaling axis via P2X7 receptors, driving macrophages toward a pro-inflammatory phenotype, while SGPL1-mediated sphingosine-1-phosphate (S1P) metabolism regulates ATP release via VRAC channels, forming a positive feedback loop (Zheng et al., 2025).

T cell subset balance is finely regulated by multiple neural signals. Glutamate spillover from synaptic clefts activates the STAT3 pathway in Th17 cells via metabotropic receptor mGluR5, while suppressing Foxp3 expression in Treg cells (Zhang et al., 2026; Marshall-Phelps and Almeida, 2024). CGRP released from nerve endings can directly act on dendritic cells, promoting their secretion of IL-23 rather than IL-12, creating a Th17-polarizing microenvironment (Zhang et al., 2024); whereas noradrenergic nerves enhance the immunosuppressive function of Treg cells via β2-adrenergic receptors (ADRB2) (Aberra et al., 2026). Synaptophysin-positive nerve endings form nanoscale “immune synapses” with T cells, and the released γ-aminobutyric acid (GABA) induces T cell metabolic reprogramming via the GABAA receptor α5 subunit, promoting glycolysis while inhibiting oxidative phosphorylation, ultimately determining the Th17/Treg differentiation fate (Bera et al., 2025; Giovannini et al., 2025).

At the level of bone metabolism regulation, norepinephrine released from sympathetic nerve endings suppresses Runx2 expression in osteoblast precursors via β2-AR signaling while activating the NFATc1 pathway in osteoclasts, constituting the core mechanism of “neurogenic bone loss” (Wang Y. et al., 2024). CGRP secreted by sensory nerves exhibits bidirectional regulation: directly promoting osteoblast differentiation via the CALCRL-RAMP1 receptor complex, while indirectly inhibiting osteoclast activity by regulating macrophage IL-10 secretion (Wang et al., 2023). Optogenetic studies further reveal that axons of basal forebrain cholinergic neurons projecting to the bone marrow release acetylcholine, which inhibits NLRP3 inflammasome assembly via α7nAChR, maintaining the dynamic balance between osteogenesis and osteoclastogenesis (Theis et al., 2025; Jaczynska et al., 2025).

In summary, the above mechanisms allow the construction of a “central (brain) → peripheral (nerve) → local (bone immune microenvironment)” three-level regulatory network model (Figure 1): the central nervous system integrates internal and external signals through hypothalamic and other nuclei, transmits them to local bone tissues via sympathetic, sensory, and parasympathetic pathways, and the various neurotransmitters and neuropeptides released from nerve endings subsequently regulate immune cell functions and bone metabolic cell activities, collectively forming the systemic regulatory basis for bone repair and remodeling.

## 4 Multi-target mechanisms of TCM in regulating the “brain-bone axis”: hierarchical interventions from central to peripheral levels

### 4.1 Central regulation: effects of kidney-tonifying herbs on the hypothalamic-pituitary axis

Kidney-tonifying herbs exhibit multi-target, multi-level regulatory advantages in modulating the hypothalamic-pituitary-adrenal/gonadal axes (HPA/HPG axes), involving fine tuning of the neuroendocrine network.

Taking icariin as an example, as the main active component of Epimedium brevicornu Maxim (Berberidaceae), its metabolites possess phosphodiesterase-5 (PDE5) inhibitory activity and may indirectly influence hypothalamic neuronal activity and modulate pulsatile GnRH secretion by enhancing the NO-cGMP signaling pathway (Xu RN. et al., 2024; Liu et al., 2025). However, it should be noted that direct experimental evidence showing that icariin metabolites cross the blood-brain barrier and act on hypothalamic GnRH neurons is currently lacking; the above mechanism is an indirect inference based on its PDE5 inhibitory activity and peripheral pharmacological effects, and requires verification in future studies using techniques such as brain microdialysis and neuron-specific calcium imaging. Animal experiments have shown that icariin significantly promotes β3-adrenoceptor-mediated adenosine release and inhibits excessive cholinergic neurotransmitter activity, a bidirectional modulation that may improve HPA axis negative feedback sensitivity and reduce glucocorticoid inhibition of the gonadal axis (Xu RN. et al., 2024; Liang X. et al., 2024).

Total flavonoids of Drynaria fortunei (Kunze) J. Sm (Polypodiaceae) primarily exert their regulatory effects through anti-oxidative stress and anti-inflammatory pathways. They inhibit the TLR4/NF-κB signaling pathway, reducing the release of pro-inflammatory cytokines such as IL-1β and TNF-α, thereby alleviating chronic stress-induced adrenal hyperplasia (Guo et al., 2024; Silva et al., 2025). In terms of gonadal axis regulation, total flavonoids of Drynaria fortunei significantly reduce malondialdehyde (MDA) content in testicular tissue of diabetic model rats, increase superoxide dismutase (SOD) activity, protect mitochondrial function in Leydig cells, and promote testosterone synthesis (Liu et al., 2025; Guo et al., 2024). This effect is similar to that of ginsenoside Rg3, which improves testicular microcirculation by upregulating endothelial nitric oxide synthase (eNOS) expression and enhances Leydig cell steroidogenic capacity (Liu et al., 2025; Silva et al., 2025).

Zuogui Pill and Yougui Pill, as classic compound formulas, exhibit synergistic bidirectional regulatory effects on the HPA/HPG axes. Zuogui Pill (左归丸) comprises Rehmannia glutinosa (Gaertn.) DC (Orobanchaceae) root (Shudihuang), Cornus officinalis Siebold & Zucc (Cornaceae) sarcocarp (Shanzhuyu), Dioscorea opposita Thunb (Dioscoreaceae) rhizome (Shanyao), Lycium barbarum L (Solanaceae) fruit (Gouqizi), Cuscuta chinensis Lam (Convolvulaceae) seed (Tusizi), *Cervus elaphus* Linnaeus (Cervidae) horn glue (Lujiaojiao), Mauremys reevesii (Gray) (Geoemydidae) shell glue (Guibanjiao), and Achyranthes bidentata Blume (Amaranthaceae) root (Niuxi). Yougui Pill (右归丸) comprises Rehmannia glutinosa root, Cornus officinalis sarcocarp, Dioscorea opposita rhizome, Lycium barbarum fruit, Cuscuta chinensis seed, *Cervus elaphus* horn glue, Eucommia ulmoides Oliv (Eucommiaceae) bark (Duzhong), Cinnamomum cassia (L.) J. Presl (Lauraceae) twig (Guizhi), Aconitum carmichaelii Debeaux (Ranunculaceae) lateral root (Fuzi), and Angelica sinensis (Oliv.) Diels (Apiaceae) root (Danggui). Components of Zuogui Pill, such as those from Rehmannia glutinosa and Cornus officinalis, attenuate glucocorticoid receptor (GR) resistance by inhibiting NLRP3 inflammasome activation, restoring the sensitivity of hypothalamic CRH neurons to cortisol negative feedback (Guo et al., 2024; Zeng et al., 2024). Yougui Pill acts synergistically through components such as astragaloside and icariin on the anterior pituitary to promote LH/FSH secretion; however, no study has directly examined the regulatory effect of Yougui Pill on the Kisspeptin/GPR54 signaling pathway, and this speculation requires further experimental validation (Liang X. et al., 2024; Zeng et al., 2024).

### 4.2 Peripheral intervention: modulation of the neurotransmitter-neuropeptide network by acupuncture and blood-activating methods

Poor fracture healing is closely associated with excessive SNS activation under stress, involving aberrant upregulation of β2-AR signaling that inhibits osteoblast function and delays bone repair (Tang et al., 2023). The kidney-tonifying and blood-activating method, as a classic TCM therapeutic principle, centers on “tonifying kidney essence” to regulate the endocrine-immune-bone metabolism axis while “activating blood and resolving stasis” to improve local microcirculation, forming a multi-target intervention strategy (Tang et al., 2023; Jiao et al., 2023). This method may improve the fracture healing microenvironment by modulating sympathetic tone and downregulating β2-AR expression or signal transduction, thereby relieving its inhibition of osteogenic differentiation. Several studies indirectly support this hypothesis: kidney deficiency syndrome models often exhibit hyperactivity of the sympathetic-adrenal medulla system, and kidney-tonifying herbs such as Drynaria fortunei and Epimedium brevicornu significantly reduce serum norepinephrine levels (Tang et al., 2023); β2-AR activation inhibits the expression of key osteogenic transcription factors such as Runx2 and Osterix via the cAMP/PKA pathway (Tang et al., 2023; Huo et al., 2023), while active components of kidney-tonifying herbs (e.g., morroniside and cornuside from Cornus officinalis) reverse this process and promote bone marrow mesenchymal stem cell differentiation toward osteoblasts (Lin et al., 2025b; Ge et al., 2024).

Fracture healing involves multi-dimensional synergy of neuropeptide-mediated bone metabolism regulation, angiogenesis, and osteoblast activation. TCM external therapies (e.g., manipulative reduction, acupuncture) and internal herbal formulas can significantly promote fracture site microenvironment repair by modulating the expression of CGRP and SP. CGRP, a neurogenic polypeptide, not only promotes osteogenic differentiation of bone marrow mesenchymal stem cells (BMSCs) by activating the cAMP/PKA/CREB signaling pathway (Hamiti et al., 2025; Qiu et al., 2024), but also upregulates the VEGF/FLK1 pathway to induce vascular endothelial cell migration and lumen formation, improving local blood supply to the fracture site (Hamiti et al., 2025; Ai and Zheng, 2025). For example, acupuncture stimulation enhances local CGRP release at the fracture site via a “peripheral-central-peripheral” neural reflex arc, promoting BMSC recruitment to the fracture site and accelerating the conversion from soft callus to hard callus (Qiu et al., 2024; Huang AS. et al., 2024). Manipulative therapy upregulates SP expression through mechanical stress stimulation of the soft tissues around the fracture; SP subsequently activates endothelial cell proliferation via the NK1R while inhibiting the release of inflammatory cytokines such as IL-1β and TNF-α, reducing local edema and pain (Hamiti et al., 2025; Cheang et al., 2024).

Regarding internal herbal formulas, active ingredients such as tetramethylpyrazine (TMP) and astragaloside exert significant “neuro-vascular-osteogenic” synergistic effects. TMP improves microcirculation at the fracture site by inhibiting platelet aggregation and reducing blood viscosity, while significantly upregulating osteogenic markers (ALP, Runx2, OPN-1) (Ai and Zheng, 2025). Its molecular mechanism involves dual activation of the VEGF/FLK1 pathway: directly promoting vascular endothelial cell generation and indirectly protecting vascular endothelial function by inhibiting inflammatory cytokines such as IL-6 and IL-1β (Ai and Zheng, 2025).

### 4.3 Immune remodeling: regulation of the bone immune microenvironment by acupuncture and moxibustion

During fracture healing, the regulation of the inflammatory response in the hematoma phase is crucial for subsequent repair stages. Recent studies have shown that “neuro-immune” coupling mechanisms play a central role in this process, and TCM (e.g., acupuncture, moxibustion) can achieve precise regulation of excessive inflammation by modulating neurotransmitter dynamics, particularly local release of acetylcholine (ACh), thereby activating the cholinergic anti-inflammatory pathway (CAP) (Guo and Russo, 2026; Khanmammadova et al., 2023; Vanden Abeele and Salzet, 2025).

At the molecular level, TCM interventions regulate neuro-immune crosstalk through multiple pathways. Acupuncture stimulation activates vagal efferent fibers, prompting T cells in immune organs such as the spleen to release ACh, which then acts on macrophages via α7nAChR to inhibit NF-κB signaling and reduce the release of pro-inflammatory cytokines such as TNF-α and IL-1β (Guo and Russo, 2026; Khanmammadova et al., 2023). Experimental data show that electroacupuncture stimulation can increase ACh levels in inflamed tissues by 40%–60% while significantly reducing NLRP3 inflammasome activation. It should be noted that these data are derived from a rat fracture hematoma model (referencing the electroacupuncture parameters in Shi JT et al., Pain, 2023 (Shi et al., 2023): 2/15 Hz dense-sparse wave, 1 mA, 30 min/session for 3 consecutive days), with ACh detection using high-performance liquid chromatography-electrochemical method. These results have been partially replicated in independent studies (e.g., multiple rodent inflammation models cited in the review by Guo X & Russo SJ, Annu Rev Immunol, 2026 (Guo and Russo, 2026)), but multi-center, cross-model consistent validation in the fracture healing field is still lacking (Vanden Abeele and Salzet, 2025; Chen et al., 2025). The thermal effect of moxibustion can induce local ATP breakdown to adenosine, which inhibits nociceptive neuron activity via A1 receptor (A1R) activation, downregulates the microglial activation marker IBA-1 and astrocyte marker GFAP, and alleviates neurogenic inflammation (Chen et al., 2025; Teoh et al., 2025).

At the cellular level, TCM regulates immune cell phenotype switching through the neuro-immune axis. Electroacupuncture promotes the recruitment of CD11b+/ICAM-1+ immune cells to the fracture site, and the β-endorphins (β-END) they secrete produce local analgesic effects by activating peripheral μ-opioid receptors (Vanden Abeele and Salzet, 2025; Shi et al., 2023). Meanwhile, norepinephrine (NE) released from sympathetic nerve endings regulates monocyte/macrophage polarization via β2-adrenergic receptors (ADR-β2), promoting the switch from pro-inflammatory M1 to anti-inflammatory M2 phenotype (Khanmammadova et al., 2023; Shi et al., 2023).

Active ingredients of TCM and compound preparations can also significantly promote macrophage polarization toward the M2 phenotype. Conditioned medium from bone marrow mesenchymal stem cells (BMSCs-CM) downregulates the pro-inflammatory markers TNF-α and iNOS, upregulates M2 markers CD206 and Arg-1, and is accompanied by increased secretion of anti-inflammatory cytokines IL-10 and TGF-β (Ji et al., 2024). This effect is closely related to activation of the TGF-β signaling pathway, which not only inhibits NLRP3 inflammasome-mediated inflammatory responses but also promotes extracellular matrix remodeling through a Smad3-dependent mechanism (Lombardo et al., 2024; Jiang et al., 2023). Notably, TCM extracts such as miR-146a-5p can inhibit the MyD88-dependent inflammatory pathway by targeting the Traf6/NF-κB signaling axis, thereby suppressing NLRP3 inflammasome assembly and activation (Huang et al., 2023; Xi et al., 2025).

In summary, TCM can promote bone repair through multi-level, multi-target regulation of the “brain-bone axis”: kidney-tonifying herbs mainly act on central endocrine links; acupuncture, manipulation, and other external therapies intervene in peripheral neurotransmitter release; and acupuncture, moxibustion, as well as some blood-activating and stasis-removing drugs exert anti-inflammatory and pro-repair effects by remodeling the bone immune microenvironment. These mechanisms are summarized in the molecular pathway diagram shown in Figure 2.

## 5 From theory to clinic: systems biology analysis of TCM orthopedic intervention strategies

### 5.1 Target differences of therapeutic principles under the three-level network framework: Stratified intervention of blood-activating, kidney-tonifying, and acupuncture/manipulation

Different TCM therapeutic principles exhibit significant differences in their target levels within the “brain-bone axis”, as compared in Table 1. Notably, the intervention windows of these principles are highly coupled with the spatiotemporal dynamics of bone repair (Figure 3). Based on the three-level regulatory network framework of the “brain-bone axis”, the three core therapeutic principles in TCM orthopedics—blood-activating and stasis-removing method, kidney-tonifying and bone-strengthening method, and acupuncture/manipulation—display distinct target levels, acting on different nodes of the neuro-endocrine-immune network.

The blood-activating and stasis-removing method primarily intervenes in the neuro-immune interaction during the fracture hematoma phase and early inflammation, with its core mechanism being the protection of the neurovascular unit (NVU). Herbs of this category (e.g., Salvia miltiorrhiza Bunge [Lamiaceae], Ligusticum striatum DC [Apiaceae]) can reduce the expression of pro-inflammatory cytokines such as TNF-α, IL-1β, and IL-6 by inhibiting the TLR4/NF-κB pathway, while promoting the release of the anti-inflammatory cytokine IL-10, thereby alleviating the neuroinflammatory cascade (Liu et al., 2023; Hou et al., 2024). This bidirectional regulation not only reduces neurotoxicity caused by excessive M1 microglial activation but also inhibits T Cell proliferation via the PD-1/PD-L1 pathway, forming an immune-tolerant microenvironment (Liu et al., 2023; Hou et al., 2024; Sun et al., 2025). At the molecular level, components such as salvianolic acid B can induce microglial M2 polarization by upregulating the SUMOylation of ANXA1, enhancing phagocytic clearance capacity and promoting tissue repair (Liang H. et al., 2024; Yang et al., 2024).

The kidney-tonifying and bone-strengthening method mainly acts on the hypothalamic-pituitary-target gland axes (HPA/HPG axes), maintaining long-term homeostasis of bone metabolism by regulating the neuroendocrine network. Kidney-tonifying herbs (e.g., Xianling Gubao, Jintiange) can activate osteogenesis-related genes (e.g., PGC-1α-mediated mitochondrial biogenesis) through multi-component synergy while inhibiting osteoclast differentiation markers (e.g., the RANKL pathway), thereby bidirectionally regulating bone formation and resorption (Jin et al., 2025; Chen et al., 2024). Further studies have shown that kidney-tonifying and bone-strengthening granules can improve bone metabolic disorders caused by sex hormone deficiency by upregulating the expression of steroid receptor coactivator-3 (SRC-3) in the bone tissue of ovariectomized rats (HUANG, 2015).

Acupuncture and manipulative therapy directly activate peripheral sensory nerve fibers (Aδ and C fibers) through physical stimulation, triggering local neuropeptide release, with CGRP being the most critical regulator (Rabanal-Rodríguez et al., 2026). CGRP possesses both potent vasodilatory and osteogenic effects: on one hand, it promotes microcirculatory blood perfusion (Kużdżał et al., 2025); on the other hand, it directly promotes bone formation by activating the CALCRL-RAMP1 receptor complex on osteoblasts (Ren et al., 2025). The “needle sensation” produced by acupuncture is essentially the activation of nociceptors via the TRPV1 channel by mechanical stimulation, triggering axon reflexes and retrograde signaling from dorsal root ganglia (Rabanal-Rodríguez et al., 2026), which can upregulate local CGRP expression by 3-5 fold while inhibiting the secretion of inflammatory cytokines such as TNF-α and IL-6 (Rabanal-Rodríguez et al., 2026), forming a localized “neuro-vascular-osteogenic” coupling regulatory network.

Evidence level caveat: The evidence grades (I-III) assigned in Table 1 reflect the authors' assessment of the existing literature and do not derive from formal GRADE or Cochrane systematic review methodology. The specific grading criteria are as follows: Grade I: includes meta-analyses or well-designed randomized controlled trials (RCTs); Grade II: includes non-randomized controlled trials, cohort studies, or case-control studies; Grade III: evidence based on animal experiments, cell experiments, or mechanistic studies. It should be particularly noted that there is currently a conspicuous absence of large-scale prospective RCTs specifically designed with “brain-bone axis” biomarkers as primary or secondary outcomes in this field; therefore, most evidence remains at Grade II-III. Especially for the “kidney-tonifying and bone-strengthening method,” although good efficacy has been observed in clinical applications, the direct causal evidence for its regulation of the “brain-bone axis” still primarily comes from animal models. Future studies need to conduct clinical research based on “brain-bone axis”-related biomarkers such as heart rate variability (reflecting autonomic balance), plasma CGRP/SP levels (reflecting sensory nerve activity), and bone turnover markers to upgrade the evidence level.

It is noteworthy that the target differences of the above therapeutic principles exhibit a dynamic evolution over the time course of bone repair: the hematoma phase is dominated by neuroinflammatory regulation; the cartilaginous callus and hard callus phases are dominated by endocrine hormones and neuro-vascular coupling; and the bone remodeling phase is dominated by neural remodeling and mechanical stress adaptation. The intervention windows of the three TCM therapeutic principles are highly coupled with this spatiotemporal dynamics (Figure 3), reflecting the TCM orthopedic concept of “treatment tailored to the phase of disease.”

## 6 Challenges and perspectives: path breakthrough from mechanism elucidation to clinical translation

Epistemological limitations and evidentiary gaps.

Several important limitations must be acknowledged regarding the framework proposed in this review.

First, the neuro-endocrine-immune network regulation of bone metabolism is highly context-dependent. The sympathetic nervous system, for example, may exert opposing effects on bone remodeling under acute versus chronic stress conditions (Ryu et al., 2024). The existing model requires further refinement to specify network response characteristics under different stress modes, inflammatory states, and metabolic conditions.

Second, the multi-component, multi-target nature of TCM formulas presents both an advantage and a challenge. In a highly integrated network such as the “brain-bone axis,” it remains unclear how multi-target interventions achieve synergy rather than antagonism, and whether unintended off-target effects occur through collateral pathways. These questions have not been fully addressed in the current literature.

Third, and most critically, the evidence base for the “brain-bone axis” regulation of bone repair in humans remains limited. The majority of mechanistic insights discussed in this review derive from rodent models, and significant transcriptomic differences exist between human and mouse hypothalamic neurons (Tadross et al., 2025), warranting caution in direct extrapolation to human bone diseases. Large-scale prospective clinical studies incorporating “brain-bone axis” biomarkers (e.g., heart rate variability, plasma neuropeptide levels) are urgently needed to elevate the evidence grade from the current predominantly preclinical level (Grade II-III) to Grade I.

Fourth, we acknowledge that the three-level model, while heuristically useful, may oversimplify the complex, non-linear, and reciprocal interactions within the neuro-endocrine-immune network. The model should be viewed as a conceptual map for organizing existing evidence and generating hypotheses, rather than a definitive representation of biological reality.

Although the “brain-bone axis” theory provides a new systems biology framework for understanding the scientific connotation of “Kidney Governs Bone,” current research still faces challenges such as spatiotemporal heterogeneity, network complexity, and clinical translation gaps. Bone repair encompasses multiple stages including the hematoma phase, cartilaginous callus phase, hard callus phase, and bone remodeling phase, and the dominant neuro-endocrine-immune network nodes differ significantly among stages; however, existing studies are mostly cross-sectional observations at a single time point, lacking dynamic tracking. The neuro-endocrine-immune network is characterized by multiple nodes, multiple levels, and non-linear interactions, but current research mostly focuses on linear analyses of a single neurotransmitter, single pathway, or single target, lacking systematic integration of the overall network structure. Moreover, existing evidence mainly comes from cell experiments and animal models, which differ significantly from the real situation of the human complex system, and there is a lack of prospective clinical studies based on “brain-bone axis” biomarkers, limiting the translation of fundamental findings to clinical applications.

Furthermore, the complexity of the theoretical model combined with the inherent characteristics of TCM interventions brings additional challenges for validation. First, the regulation of bone metabolism by the sympathetic nervous system may be context-dependent: the sympathetic output pattern and its net effect on bone remodeling may differ significantly between acute and chronic stress states, and the existing model needs to further refine the network response characteristics under different stress modes. Second, the multi-component, multi-target nature of TCM formulas is both an advantage and a challenge: in the highly integrated “brain-bone axis” network, how does multi-target intervention achieve synergy rather than antagonism? Could it produce off-target effects through unintended pathways? These are core questions in the mechanism study of TCM formulas that have not yet been fully answered. Future research can achieve breakthroughs in the following directions.

1. Optogenetic and chemogenetic manipulation: Using optogenetic and chemogenetic techniques to precisely manipulate the activity of specific brain region (e.g., arcuate nucleus, ventromedial hypothalamus) neuronal subpopulations, combined with dynamic observation of bone repair, to verify how changes in central neuronal activity mediate the bone repair-promoting effects of TCM, thereby establishing at a causal level the central mechanism of “brain-bone axis” regulation.
2. Multi-omics integration analysis: Using multi-omics technologies such as single-cell sequencing, spatial transcriptomics, and metabolomics to map the spatiotemporal dynamic landscape of the “brain-bone axis” under TCM intervention, revealing the molecular evolution patterns and network interactions of key cell subpopulations (e.g., neural stem cells, immune cells, osteolineage cells) at different stages of bone repair.
3. Organoid and microfluidic chip platforms: Constructing “neuro-bone” organoid co-culture models and microfluidic chip platforms to simulate the nerve-innervated bone repair microenvironment, for high-throughput screening of TCM active ingredients with the potential to regulate the “brain-bone axis” and to explore their mechanisms of action, providing novel *in vitro* models for drug development.
4. Biomarker-based clinical studies: Conducting TCM syndrome differentiation and efficacy prediction studies based on “brain-bone axis” biomarkers. Select indicators reflecting sympathetic/vagal tone such as heart rate variability, plasma neuropeptide (CGRP, SP) levels, and bone metabolic markers, explore their associations with TCM syndrome types (e.g., kidney deficiency syndrome, blood stasis syndrome), and establish a “syndrome-neural phenotype-efficacy response” prediction model, promoting the transformation of TCM orthopedics from empirical medicine to a precision medicine paradigm.

## 7 Conclusion: TCM orthopedics moving toward systems medicine

As a core proposition of TCM theory, the modern scientific connotation of “Kidney Governs Bone” has transcended the single framework of the traditional “kidney-bone” endocrine axis and is profoundly reflected in the systemic regulation of the “neuro-endocrine-immune network.” Focusing on the core proposition that “the therapeutic efficacy of TCM’s “Kidney Governs Bone” can be partially explained by its systemic regulation of the neuro-endocrine-immune network,” and based on the three-level regulatory network model of the “brain-bone axis,” this paper systematically reviews the synergistic roles of the central hub (hypothalamus-pituitary), descending pathways (sympathetic, sensory, and parasympathetic nerves), and terminal effector (bone immune microenvironment) in bone repair. It further integrates evidence revealing that TCM achieves dynamic regulation of the “brain-bone axis” through multi-target pathways including modulation of central endocrine links, intervention in peripheral neurotransmitters and neuropeptides, and remodeling of the bone immune microenvironment. This interdisciplinary research direction not only provides a modern scientific interpretation of the “Kidney Governs Bone” theory but also marks a paradigm shift of TCM orthopedics from empirical medicine toward systems medicine, opening up an important theoretical breakthrough for the modernization and internationalization of the discipline.
