# Supplementary material for: A modern reinterpretation of the “kidney governs bone” theory: systemic regulation of the neuro-endocrine-immune network via the brain-bone axis and traditional chinese medicine intervention strategies
Source: Front Pharmacol. 2026 Jul 29;17:1862657. doi: 10.3389/fphar.2026.1862657 (PMC13461624; doi:10.3389/fphar.2026.1862657)
Supplement: Supplementary file 1 [file Table1.docx]

**Supplementary Table S1.**

**Table S1 Protective Effects of Tetramethylpyrazine in Various Disease Models**

| **Disease model** | **Organ/system** | **Key molecular targets** | **Protective effect** | **Evidence level** | **References** |
| --- | --- | --- | --- | --- | --- |
| Sepsis-induced liver injury | Liver | TFR1↓, Atf3↓, GPX4↑, Nrf2↑, FSP1↑ | Inhibits ferroptosis | +++ | [20] |
| Chronic kidney disease | Kidney | IGF2BP1-Odc1 interaction↓ | Inhibits ferroptosis | ++ | [21] |
| Amyotrophic lateral sclerosis | CNS | GABAAR (predicted) | Slows functional decline | +++ | [23] |
| Cerebral ischemia-reperfusion | Brain | p-JNK↓ | Reduces neuronal apoptosis | +++ | [68] |
| Coronary artery calcification | Cardiovascular | Caspase-3/GSDME↓ | Inhibits pyroptosis | ++ | [47] |
| Migraine | Cerebral vasculature | Nrf2 axis | Restores GSH/ATP | ++ | [16] |
| Bone fracture repair | Bone | Osteo-angiogenesis↑ | Promotes healing | ++ | [26] |
| Blood-brain barrier injury | CNS | miR-297c-5p↓, occludin↑ | Improves BBB integrity | ++ | [19] |

Evidence level: +++ validated in vivo or in clinical trials; ++ validated in vitro or in other disease models in vivo; + predicted based on pathways.
